# Associations of Harsh Physical Punishment and Child Maltreatment in Childhood With Antisocial Behaviors in Adulthood

**DOI:** 10.1001/jamanetworkopen.2018.7374

**Published:** 2019-01-25

**Authors:** Tracie O. Afifi, Janique Fortier, Jitender Sareen, Tamara Taillieu

**Affiliations:** 1 Department of Community Health Sciences, University of Manitoba, Winnipeg, Manitoba, Canada; 2 Department of Psychiatry, University of Manitoba, Winnipeg, Manitoba, Canada; 3 Department of Psychology, University of Manitoba, Winnipeg, Manitoba, Canada

## Abstract

**Importance:**

Inquiry into what childhood experiences are associated with antisocial behaviors in adulthood is necessary for prioritizing and informing efforts for effective prevention.

**Objective:**

To examine whether harsh physical punishment in the absence of child maltreatment and child maltreatment with and without harsh physical punishment are associated with antisocial behaviors in adulthood.

**Design, Setting, and Participants:**

Cross-sectional study using data on the general US population obtained from the National Survey on Alcohol and Related Conditions Wave 3 from April 2012 to June 2013. Participants were civilian, noninstitutionalized adults 18 years and older. This study used a multistage probability sampling design (response rate, 60.1%). Data were analyzed from January 25 to November 27, 2018.

**Exposures:**

Harsh physical punishment included pushing, grabbing, shoving, slapping, and hitting. Child maltreatment included physical abuse, sexual abuse, emotional abuse, physical neglect, emotional neglect, and exposure to intimate partner violence.

**Main Outcomes or Measures:**

Lifetime antisocial personality disorder behaviors since age 15 years were assessed using the Alcohol Use Disorder and Associated Disabilities Interview Schedule-5 based on *Diagnostic and Statistical Manual of Mental Disorders (Fifth Edition)* criteria.

**Results:**

The number of study participants was 36 309, with 15 862 men (weighted percentage, 48.1%) and 20 447 women (weighted percentage, 51.9%); mean (SE) age was 46.54 (0.19) years. The prevalence of harsh physical punishment and child maltreatment was 18.1% and 46.7%, respectively. Harsh physical punishment only (adjusted β, 0.62; 95% CI, 0.50-0.75), child maltreatment only (adjusted β, 0.65; 95% CI, 0.60-0.69), and harsh physical punishment and child maltreatment (adjusted β, 1.46; 95% CI, 1.38-1.54) were associated with adult antisocial behaviors. It is estimated that harsh physical punishment and/or child maltreatment might account for approximately 45.5% of antisocial behaviors among men in the United States and 47.3% antisocial behaviors among women in the United States.

**Conclusions and Relevance:**

Harsh physical punishment and child maltreatment appear to be associated with adult antisocial behaviors. Preventing harsh physical punishment and child maltreatment in childhood may reduce antisocial behaviors among adults in the United States.

## Key Points

**Question:**

Are harsh physical punishment in the absence of child maltreatment and child maltreatment with and without harsh physical punishment in childhood associated with antisocial behaviors in adulthood?

**Findings:**

In this cross-sectional study using nationally representative data on 36 309 adults, harsh physical punishment in the absence of child maltreatment and child maltreatment that they had experienced were associated with antisocial behavior. Together, these issues are estimated to account for 45.5% and 47.3% of antisocial behaviors among men and women, respectively, in the United States.

**Meaning:**

Preventing harsh physical punishment and child maltreatment may be associated with decreases in adult antisocial behaviors in the general population.

## Teaser

This cross-sectional study examines whether harsh physical punishment, child maltreatment, and concomitant occurrence experienced during childhood are associated with antisocial behaviors in adulthood.

## Introduction

Nationally representative data from the United States indicate that 80% of children have been spanked by the time they are in kindergarten.^1^ Spanking is typically defined as hitting a child on the buttocks with an open hand. When looking at the collective evidence from meta-analyses, it is found that spanking or other physical punishment is associated with an increased likelihood of aggression, lower moral internalization, antisocial behavior, externalizing problems, internalizing problems, poorer mental health, and negative relationships with parents.^2,3^ Messages have appeared on major news networks, in print, online, and on television encouraging people to stop spanking, slapping, hitting, and other forms of harsh physical punishment.^4^ However, despite this evidence, there are still opinions in the media on major news networks stating that societal problems are a result of not spanking and hitting children enough.^5^

Studies have examined physical punishment and antisocial outcomes mostly within childhood or adolescence.^6^ For example, studies have found that spanking was associated with increased aggression,^7^ behavioral problems,^8^ antisocial behavior,^9,10^ and externalizing behaviors during childhood.^1,11,12^ Many of the previous studies have focused on the early developmental period of preschool or young, school-aged children, with the time between assessments varying across studies. Less is known about how physical punishment in childhood is associated with antisocial behaviors in adulthood. Most recently, a study using samples from Asia, Europe, and North America examined harsh physical punishment, including spanking, slapping, smacking or swatting, grabbing, shaking, hitting with an object, and/or using soap or hot sauce on the child’s tongue, and found that these discipline methods were associated with antisocial behavior in adulthood, including hitting a person outside of their family, physically attacking someone, and stealing money.^6^ In a cross-sectional study, harsh physical punishment (ie, pushing, grabbing, shoving, slapping, and hitting) without experiencing more severe child maltreatment was associated with increased odds of many mental disorders in adulthood, including antisocial personality disorder, after adjusting for sociodemographic variables and household dysfunction.^13^ Other studies looking at the association between physical punishment and adult antisocial outcomes have also been limited by narrow definitions of antisocial behavior^6^ or the inability to examine physical punishment separately from child physical abuse.^14^

It is important that research be able to examine the independent effects of spanking or harsh physical punishment from more severe child maltreatment and that significant findings not be owing to confounding or unmeasured effects of child maltreatment. For example, previous research found that, after adjusting for physical and emotional abuse, spanking was associated with several poor adult outcomes, including suicide attempts, moderate to heavy drinking, and drug use.^15^ Research examining harsh physical punishment only and child maltreatment with or without harsh physical punishment found that, as the violence increases, generally so do poor mental^16^ and physical^17^ health outcomes in adulthood. An additional limitation of the existing literature is the lack of examination of sex differences in the association between physical punishment, child maltreatment, and antisocial behaviors. It may be that many studies are not adequately powered to test for sex interaction effects or to run sex-stratified models. In addition, many studies are limited by the use of convenience general population samples or at-risk samples with only a few investigations using nationally representative data.

Antisocial features of antisocial personality disorder as defined in the *Diagnostic and Statistical Manual of Mental Disorders (Fifth Edition)* (*DSM-5*) include failure to conform to the law, lack of ethical behavior, absence of concern for others while being deceitful, and irresponsible, manipulative, and risk-taking behaviors.^18^ Adults who demonstrate antisocial behaviors may experience many difficulties in developing and maintaining positive relationships, have trouble sustaining successful employment, and have problems with the law, among other challenges.^19,20,21^ Antisocial behaviors are generally found to be more prevalent among men compared with women, again making the examination of sex differences an important consideration.^22,23,24^ Harsh physical punishment and child maltreatment are likely associated with antisocial behaviors among men and women in the general population in the United States—the extent, however, is unknown. Inquiry into what childhood experiences are associated with antisocial behaviors in adulthood is necessary for prioritizing and informing efforts for effective violence prevention and to refute unfounded opinions in the media.

The present study addresses some of the limitations in the existing literature by the use of nationally representative data from the United States, examination of sex differences, consideration for temporal order by using retrospective self-reports of childhood harsh physical punishment and child maltreatment experiences with measures of antisocial behaviors in adulthood, and assessments of *DSM-5* antisocial personality disorder criteria. The objectives of the present study were to determine (1) if sex moderates the association between harsh physical punishment and/or child maltreatment and antisocial behaviors (if so, sex-stratified analyses are required); (2) if harsh physical punishment alone is associated with antisocial behaviors after adjusting for sociodemographic variables; (3) if the more severe childhood adversity (ie, child maltreatment without harsh physical punishment and child maltreatment with harsh physical punishment) compared with harsh physical punishment only is associated with antisocial behaviors after adjusting for sociodemographic variables; and (4) what proportion of antisocial behaviors among adults in the general population of the United States is associated with harsh physical punishment and child maltreatment.

## Methods

### Data and Sample

Data were obtained from the National Survey on Alcohol and Related Conditions Wave 3 (NESARC-III) collected from April 2012 to June 2013 using in-person interviews (N = 36 309). A multistage probability sampling design was used to randomly select civilian, noninstitutionalized adults representative of the US population (response rate, 60.1%). More information on the NESARC-III can be found in previous publications.^25,26^ The research protocol, including providing written informed consent, was approved by the Westat Institutional Review Board and the Combined Neuroscience Institutional Review Board of the National Institutes of Health.^27^ The University of Manitoba Health Research Ethics Board, Winnipeg, Manitoba, Canada, provided ethical approval for the data analysis. Data were analyzed from January 25 to November 27, 2018. This study followed the Strengthening the Reporting of Observational Studies in Epidemiology (STROBE) reporting guideline.

### Measurement

#### Harsh Physical Punishment and Child Maltreatment

Seven types of child maltreatment before the age of 18 years, including harsh physical punishment, physical abuse, sexual abuse, emotional abuse, emotional neglect, physical neglect, and exposure to intimate partner violence (EIPV), were measured to create a composite, 4-level harsh physical punishment and child maltreatment variable. Questions to assess child maltreatment were adapted from the Adverse Childhood Experiences study,^28,29^ a subset of questions from the Conflict Tactics scale,^30,31^ and the Childhood Trauma Questionnaire.^32^ Conventional coding with recommended cutoffs for assessing child maltreatment were used.

Harsh physical punishment was assessed by asking respondents, “Before you were 18, how often did a parent/other adult living in your home push, grab, shove, slap, or hit you?” Respondents who reported a response of sometimes or more on a 5-point ordinal scale (never, almost never, sometimes, fairly often, and very often) were categorized as having experienced harsh physical punishment. A dichotomous any child maltreatment variable was created to determine the presence or absence of any of the following child maltreatment types: physical abuse, sexual abuse, emotional abuse, emotional neglect, physical neglect, and EIPV. These individual child maltreatment types were coded as follows. Respondents who reported any response other than never on the question, “How often did a parent or other adult living in your home hit you so hard that you had marks or bruises or were injured?” were categorized as having experienced physical abuse. Respondents were categorized as having experienced sexual abuse if they ever experienced any unwanted sexual touching or fondling and/or attempted or completed sexual intercourse by an adult or other person. Respondents who reported experiencing a parent or other adult living in the home swear at, insult, say hurtful things, threaten to hit, or throw something at the respondent or act in any other way that made the respondent afraid of being physically hurt fairly often or very often were categorized as having experienced emotional abuse. Emotional neglect was defined as having a summed score of 15 or greater on 5 reverse-coded questions on a 5-point ordinal scale (never true, rarely true, sometimes true, often true, and very often true) including how often the respondent felt part of a close-knit family, felt the family was a source of strength and support, and felt that a family member wanted the respondent to succeed, made the respondent feel important or special, and believed in the respondent.^28,29,33,34^ Respondents who reported ever having been left alone or unsupervised before the age of 10 years or going without necessary clothing, school supplies, food, or medical treatment were categorized as having experienced physical neglect.

Exposure to intimate partner violence was determined by asking respondents whether their mother’s partner had pushed, grabbed, slapped, or thrown something at their mother (sometimes, fairly often, or very often); kicked, bit, or hit their mother with a fist or something hard (sometimes, fairly often, or very often); ever repeatedly hit their mother for at least a few minutes (any response other than never); or had ever threatened her with a knife or gun or used a knife or gun to hurt her (any response other than never). A composite 4-level harsh physical punishment and child maltreatment variable was then computed by summing the harsh physical punishment and composite any child maltreatment variables (no harsh physical punishment and no child maltreatment, harsh physical punishment only, child maltreatment only, and harsh physical punishment and child maltreatment).

#### Antisocial Personality Disorder Symptoms

Lifetime antisocial personality disorder symptoms since age 15 years were assessed using the Alcohol Use Disorder and Associated Disabilities Interview Schedule-5 based on *DSM-5* criteria.^18^ Table 1 presents the coding of 6 antisocial behavior categories, including (1) failure to conform to social norms with respect to lawful behaviors as indicated by repeatedly performing acts that are grounds for arrest; (2) deceitfulness, as indicated by repeated lying, use of aliases, or conning others for personal profit or pleasure; (3) impulsivity or failure to plan ahead; (4) irritability and aggressiveness, as indicated by repeated physical fights or assaults; (5) reckless disregard for safety of self or others; and (6) consistent irresponsibility, as indicated by repeated failure to sustain consistent work behavior or honor financial obligations. A continuous variable of antisocial behaviors was computed (0-6 categories). A seventh category assessing lack of remorse was not included in the analysis because these items were not asked of the whole sample and were limited to respondents with at least 3 of the previously listed categories.

**Table 1.** Antisocial Personality Disorder Symptoms Since Age 15 Years

Antisocial Personality Disorder Symptoms	No. of Items	Questionnaire Items	Coding
Antisocial symptom 1: failure to conform to social norms with respect to lawful behaviors as indicated by repeatedly performing acts that are grounds for arrest	12	Destroy or damage someone else’s property (eg, car, home); start fire on purpose to destroy someone else’s property or just to see it burn; steal something from someone/someplace when no one was around; forge a check or any other document; break into someone else’s house, building, or car; shoplift; steal from someone directly; make money illegally, such as selling stolen property or selling drugs; use someone’s credit card without their permission; steal online or scam over the telephone; do something you could have been arrested for, regardless of whether caught or not; harass, threaten, or blackmail someone	Yes on ≥1 item
Antisocial symptom 2: deceitfulness, as indicated by repeated lying, use of aliases, or conning others for personal profit or pleasure	4	Have a time when you lied a lot, other than to avoid being hurt; use a false or made-up name or alias; scam or con someone for money to avoid responsibility or just for fun; often exaggerate, change facts, or stretch truth to make a better story	Yes on ≥1 item
Antisocial symptom 3: impulsivity or failure to plan ahead	6	Make spur of the moment decisions, eg, quitting school, moving, or changing jobs; travel from place to place for ≥1 mo without advance plans or without knowing how long would be gone or where would work; have time lasting ≥1 mo when had no regular place to live; have time lasting ≥1 mo when lived with others because did not have own place to live; tend to do things without thinking about them very much, just to keep from being bored; tend to do things without thinking about what would happen as a result	Yes on ≥1 item
Antisocial symptom 4: irritability and aggressiveness, as indicated by repeated physical fights or assaults	8	Often bully or push people around or try to make them afraid of you; force someone to engage in any sexual activity with you against their will; get into a lot of fights that you started; physically hurt another person in any other way on purpose; get into a fight that came to swapping blows with someone like a husband, wife, girlfriend, or boyfriend; use a weapon like a stick, knife, or gun in a fight; hit someone so hard that you injured them or they had to see a doctor; hurt or be cruel to an animal or pet on purpose	Yes on ≥1 item
Antisocial symptom 5: reckless disregard for safety of self or others	4	Often do risky or dangerous things, not caring about consequences; do things that could easily have hurt you or someone else, like speeding or driving or using heavy machinery while drunk or high; have unprotected sex other than with spouse or in committed relationship; have driver’s license suspended or revoked for moving violations	Yes on ≥1 item
Antisocial symptom 6: consistent irresponsibility, as indicated by repeated failure to sustain consistent work behavior or honor financial obligations	4	Often cut class, not go to class, or go to school and leave without permission^a^; have a time when absent from work a lot, other than when sick, caring for someone who was sick, or on military duty; more than once quit a job without knowing where would find another one; fail to pay off debts, eg, moving to avoid rent, not making payments on loan or mortgage, failing to pay alimony or child support	Yes on ≥1 item
Antisocial symptom 7: lack of remorse, as indicated by being indifferent to or rationalizing having hurt, mistreated, or stolen from another^b^	3	Since time when destroyed property, stole something, or mistreated/harmed another person, have you regretted doing these things or wished they never happened?^c^ Did you feel you had a right to do any of these things (destroy property, steal something, mistreat/harm another person)? Ever feel other people deserved what they got?	Yes on ≥1 item

^a^ Since age 1 year.

^b^ Only asked of a subset of the population who reported at least 3 experiences since age 15 years, or reported at least 1 experience when they destroyed property, stole something, or mistreated/harmed another person.

^c^ No is the symptomatic response; item was then reverse coded.

#### Covariates

Several sociodemographic variables were adjusted for in the statistical models. Self-reported sociodemographic covariates included in the study were age, marital status, race/ethnicity, household income, and educational level.

### Statistical Analysis

Missing data were noted, and all variables were determined to have less than 1% missing with the exception of the continuous antisocial behavior variable, with 11.4% missing cases. Cross-tabulations for descriptive statistics, linear regressions, and population-attributable fractions (PAFs) were conducted with a 95% level of statistical significance. Statistical weights were applied to correct for oversampling and ensure that NESARC-III data were representative of the US population. To account for the complex sampling design, Taylor series linearization was used as a variance estimation technique. First, descriptive statistics were computed. Second, interaction effects were computed to determine whether sex moderated the associations between harsh physical punishment and/or child maltreatment and antisocial behaviors. Third, linear regressions were computed to examine the association between harsh physical punishment and child maltreatment and antisocial symptoms, adjusting for sociodemographic variables. Fourth, differences between harsh physical punishment only, child maltreatment only, and harsh physical punishment and child maltreatment on antisocial behaviors in adulthood were tested by changing the reference group and rerunning the models.

Fifth, PAFs were computed to estimate what proportion of antisocial behaviors in adulthood in the general population in the United States might be associated with experiences of harsh physical punishment and/or child maltreatment. The PAFs estimate the proportion of the outcomes in the population that might be reduced if the exposure were eliminated.^35^ An assumption of PAFs is that the association between the independent and dependent variable is causal. This assumption could not be determined owing to the nature of the cross-sectional data. For the linear regression models, we tested the assumptions of normality, linearity, and homoscedasticity of residuals. The *P* value for the residuals was statistically significant, which is expected with a large sample size.^36^ However, plotted data were close to normal and did not appear to indicate that any assumptions were substantively violated. To test the assumptions, we used the Kernel density plot as well as qnorm plots and Shapiro-Wilk test; *P* values were calculated using a 1-sided test, and the level of significance was *P* < .001. Stata version 15 (StataCorp) was used for statistical analysis.

## Results

The number of participants in the present study was 36 309, with 15 862 men (weighted percentage, 48.1%) and 20 447 women (weighted percentage, 51.9%) (mean [SE] age, 46.54 [0.19] years). The prevalence of harsh physical punishment was 18.1% (18.5% among men and 17.7% among women). The prevalence of any child maltreatment was 46.7% (46.6% among men and 46.8% among women). Table 2 provides the descriptive information on the distribution of the respondents’ sociodemographic information among those who experienced no harsh physical punishment or child maltreatment, harsh physical punishment only, child maltreatment only, and both harsh physical punishment and child maltreatment. Statistically significant (*P* < .05) sex interaction effects were found for child maltreatment only and child maltreatment and harsh physical punishment such that women reported significantly fewer antisocial behaviors compared with men. The remaining models were run sex stratified.

**Table 2.** Weighted Prevalence of Harsh Physical Punishment and Child Maltreatment by Sociodemographic Variables

Sociodemographic Variable	% (95% CI)			
	No CM or HPP	HPP Only	CM Only	CM and HPP
Sex				
Men	50.67 (49.41-51.93)	2.78 (2.45-3.14)	30.81 (29.81-31.84)	15.74 (14.87-16.64)
Women	51.54 (50.35-52.73)	1.76 (1.52-2.03)	30.60 (29.67-31.56)	16.10 (15.28-16.95)
Age, y				
18-29	55.63 (54.04-57.20)	1.52 (1.18-1.96)	30.71 (29.44-32.00)	12.15 (11.08-13.31)
30-39	50.81 (49.18-52.44)	2.07 (1.71-2.51)	31.05 (29.68-32.44)	16.07 (15.01-17.19)
40-49	47.64 (45.99-49.29)	2.12 (1.70-2.64)	31.34 (29.97-32.75)	18.90 (17.69-20.18)
50-59	47.47 (45.67-49.27)	2.78 (2.27-3.40)	30.81 (29.38-32.29)	18.95 (17.52-20.46)
≥60	52.60 (50.81-54.38)	2.72 (2.30-3.21)	29.94 (28.65-31.26)	14.74 (13.66-15.89)
Marital status				
Married/common law	51.30 (50.15-52.46)	2.46 (2.18-2.78)	30.23 (29.36-31.11)	16.01 (15.15-16.90)
Widowed/divorced/ separated	47.62 (46.01-49.24)	2.23 (1.89-2.62)	31.29 (29.91-32.69)	18.86 (17.69-20.09)
Single/never married	53.68 (52.17-55.19)	1.73 (1.41-2.11)	31.43 (30.04-32.85)	13.16 (12.20-14.19)
Race/ethnicity				
White	51.85 (50.53-53.16)	2.28 (2.00-2.60)	30.05 (29.09-31.03)	15.82 (14.94-16.75)
Black	47.89 (45.70-50.08)	3.23 (2.70-3.87)	30.69 (29.06-32.36)	18.20 (16.83-19.65)
American Indian/Alaska Native	39.43 (34.17-44.94)	1.52 (0.67-3.43)	31.80 (27.01-37.00)	27.25 (23.55-31.31)
Asian/Native Hawaiian/other Pacific Islander	54.92 (51.90-57.90)	2.47 (1.68-3.60)	31.91 (28.89-35.09)	10.71 (9.27-12.33)
Hispanic	50.18 (48.56-51.79)	1.33 (1.02-1.74)	33.08 (31.68-34.51)	15.41 (14.47-16.40)
Household income, $				
0-19 999	48.11 (46.44-49.78)	1.75 (1.41-2.16)	32.35 (30.90-33.83)	17.80 (16.64-19.02)
20 000-39 999	49.87 (48.32-51.42)	2.01 (1.69-2.38)	31.60 (30.33-32.91)	16.51 (15.57-17.50)
40 000-69 999	50.46 (49.04-51.88)	2.25 (1.83-2.76)	30.73 (29.60-31.88)	16.57 (15.39-17.82)
≥70 000	54.36 (52.94-55.76)	2.74 (2.36-3.17)	29.02 (27.81-30.26)	13.89 (12.92-14.91)
Education				
Less than high school	47.74 (45.41-50.07)	1.46 (1.09-1.95)	32.65 (30.91-34.44)	18.16 (16.51-19.93)
High school	49.64 (47.89-51.38)	2.46 (2.00-3.01)	31.29 (29.90-32.72)	16.61 (15.50-17.79)
Some college	48.91 (47.28-50.55)	2.65 (2.24-3.13)	30.56 (29.32-31.82)	17.88 (16.77-19.05)
Completed postsecondary degree	54.38 (53.03-55.72)	2.15 (1.85-2.50)	29.78 (28.69-30.89)	13.69 (12.81-14.61)

Abbreviations: CM, child maltreatment; HPP, harsh physical punishment.

The findings for the associations between harsh physical punishment and child maltreatment and antisocial symptoms are reported in Table 3. Harsh physical punishment only (adjusted β, 0.62; 95% CI, 0.50-0.75), child maltreatment only (adjusted β, 0.65; 95% CI, 0.60-0.69), and harsh physical punishment and child maltreatment (adjusted β, 1.46; 95% CI, 1.38-1.54) were associated with adult antisocial behaviors. In a sensitivity analysis when further adjustment of models for conduct disorder symptoms were counted in childhood, findings with regard to statistical significance remained the same.

**Table 3.** Associations Between HPP and CM and Number of Antisocial Symptoms, Stratified by Sex

Population	No HPP, No CM	Adjusted β (95% CI)^a^^,^^b^			Differences Across Mutually Exclusive Groups^c^
		HPP Only	CM Only	HPP and CM	
Men	1 [Reference]	0.69 (0.50-0.88)	0.71 (0.63-0.79)	1.76 (1.64-1.88)	HPP only = CM only
					HPP only <HPP and CM
					CM only <HPP and CM
Women	1 [Reference]	0.54 (0.35-0.74)	0.59 (0.53-0.66)	1.21 (1.12-1.30)	HPP only = CM only
					HPP only <HPP and CM
					CM only <HPP and CM
Total sample	1 [Reference]	0.62 (0.50-0.75)	0.65 (0.60-0.69)	1.46 (1.38-1.54)	HPP only = CM only
					HPP only <HPP and CM
					CM only <HPP and CM

Abbreviations: CM, child maltreatment; HPP, harsh physical punishment.

^a^ Adjusted for age, race/ethnicity, marital status, total household income, and educational level.

^b^ *P* < .001.

^c^ Determined by changing the reference group and rerunning the models. The equal symbol indicates nonsignificant differences between the 2 groups.

When examining any antisocial behaviors (none vs ≥1), it is estimated that eliminating all harsh physical punishment and child maltreatment may reduce any antisocial behavior. The PAF for harsh physical punishment and child maltreatment and antisocial behaviors for men was 45.5% (95% CI, 42.1%-48.9%); women, 47.3% (95% CI, 44.0%-50.6%); and the total sample, 46.7% (95% CI, 44.4%-48.8%) in the general population of the United States. Estimates of PAF were adjusted for age, race/ethnicity, marital status, total household income, and educational level. Total sample PAF estimates were additionally adjusted for sex.

## Discussion

Several novel findings from the present study were noted. First, sex moderated the association between child maltreatment only and child maltreatment and harsh physical punishment, with associations indicating that the effect size for women compared with men is lower. Second, harsh physical punishment only, child maltreatment only, and harsh physical punishment and child maltreatment were all associated with antisocial behaviors in adulthood among men and women. Third, the associations between harsh physical punishment only and antisocial behavior and child maltreatment only and antisocial behavior were not significantly different. However, the effect size was significantly larger for the associations between experiencing both harsh physical punishment and child maltreatment and antisocial behavior. Fourth, among women, 47.3% of any antisocial behaviors may be associated with harsh physical punishment and/or child maltreatment; for men, 45.5% may be associated with harsh physical punishment and/or child maltreatment.

The findings from the present study are consistent with those of previous research that indicate that harsh physical punishment in childhood is associated with antisocial behavior in adulthood.^6^ However, the findings from the present study also extend the knowledge. Although antisocial behaviors have been found to be more prevalent among men,^22,23^ our findings suggest that the associations between harsh physical punishment and child maltreatment and antisocial behaviors are similar for men and women. Harsh physical punishment in childhood, including pushing, grabbing, shoving, slapping, and hitting without more severe child maltreatment, was associated with antisocial behaviors in adulthood. Significant differences were not found between harsh physical punishment only and child maltreatment only with regard to the association with antisocial behaviors among men and women. However, the association with antisocial behavior was larger among those who experienced both harsh physical punishment and child maltreatment. This finding indicates that the more violence a child experiences in childhood, the greater the association with antisocial behaviors in adulthood. These findings suggest that violence prevention strategies need to include prevention of pushing, grabbing, shoving, slapping, and hitting children along with strategies to prevent more severe child maltreatment.

The PAFs also add to our current state of knowledge. These findings indicate that 47.3% of any antisocial behaviors in women and 45.5% in men in the general population in the United States may be associated with experiencing harsh physical punishment and/or child maltreatment in childhood. Theoretically, this estimate indicates that, if all experiences of harsh physical punishment and child maltreatment could be eliminated, a reduction of antisocial behaviors of 47.3% among women and 45.5% among men in the general population in the United States may be noted.

### Limitations

Several limitations of the present study should be considered when interpreting the findings. First, the data were cross-sectional. Although data from childhood were collected retrospectively, other research has indicated that this method has validity.^37^ However, disentangling the experiences of harsh physical punishment from child maltreatment is difficult using survey data. Second, although a temporal order was established using the present design with harsh physical punishment and child maltreatment occurring before the antisocial behaviors, a causal relationship cannot be inferred. Thus, an assumption about attributable fractions is that the association between the exposure and outcome are causal, which cannot be established with our data. As well, antisocial behaviors were measured using self-reported data. This type of reporting is a limitation because individuals may be less likely to report socially undesirable or even criminal behavior, which may account for some missing data. Ideally, an alternative data source would be used to confirm antisocial behavior; however, this was not possible for these data. In addition, not all respondents were asked about the lack of remorse for antisocial behaviors and were not included in the models.

## Conclusions

Investments into effective prevention efforts to eliminate harsh physical punishment of children (pushing, grabbing, shoving, slapping, and hitting) and child maltreatment (physical abuse, sexual abuse, emotional abuse, emotional neglect, physical neglect, and EIPV) should be a public health priority. If we are able to prevent harsh physical punishment and child maltreatment in childhood, we may see reductions in antisocial behaviors among men and women in the general population in the United States.
